# Factor structure of the Hospital Anxiety and Depression Scale in Japanese psychiatric outpatient and student populations

**DOI:** 10.1186/1477-7525-7-42

**Published:** 2009-05-17

**Authors:** Tomomi Matsudaira, Hiromi Igarashi, Hiroyoshi Kikuchi, Rikihachiro Kano, Hiroshi Mitoma, Kiyoshi Ohuchi, Toshinori Kitamura

**Affiliations:** 1 Department of Clinical Behavioural Sciences (Psychological Medicine), Kumamoto University, Graduate School of Medical Sciences, 1-1-1 Honjo, Kumamoto, Kumamoto, Japan 860-8556; 2 Graduate School of Clinical Psychology, Tokyo International University, 2-6-1 Nishiwaseda, Shijuku, Tokyo, Japan 169-0051; 3 Mitoma Clinic, 2-5-12 Shin-ohe, Kumamoto, Kumamoto, Japan 862-0972; 4 Heartful Clinic, 5-10-23 Hotakubo, Kumamoto, Kumamoto, Japan 862-0926

## Abstract

**Background:**

The Hospital Anxiety and Depression Scale (HADS) is a common screening instrument excluding somatic symptoms of depression and anxiety, but previous studies have reported inconsistencies of its factor structure. The construct validity of the Japanese version of the HADS has yet to be reported. To examine the factor structure of the HADS in a Japanese population is needed.

**Methods:**

Exploratory and confirmatory factor analyses were conducted in the combined data of 408 psychiatric outpatients and 1069 undergraduate students. The data pool was randomly split in half for a cross validation. An exploratory factor analysis was performed on one half of the data, and the fitness of the plausible model was examined in the other half of the data using a confirmatory factor analysis. Simultaneous multi-group analyses between the subgroups (outpatients vs. students, and men vs. women) were subsequently conducted.

**Results:**

A two-factor model where items 6 and 7 had dual loadings was supported. These factors were interpreted as reflecting anxiety and depression. Item 10 showed low contributions to both of the factors. Simultaneous multi-group analyses indicated a factor pattern stability across the subgroups.

**Conclusion:**

The Japanese version of HADS indicated good factorial validity in our samples. However, ambiguous wording of item 7 should be clarified in future revisions.

## Background

The Hospital Anxiety and Depression Scale (HADS) [1] is a self-report screening instrument for negative moods. The HADS was developed to identify people with physical illness who present anxiety and depressive disorders. To discern somatic symptoms of anxiety and depression from those caused by physical illness, the HADS taps only the affective and cognitive aspects of anxiety and depression. The HADS consists of 14 items; the anxiety (HADS-A) and depression (HADS-D) subscales each include 7 items. The conciseness of the HADS allows a high degree of usability in both clinical and research settings.

The reliability and validity of the HADS has been well established [2,3]. However, previous studies have reported inconsistent factor structures. Earlier studies, which used exploratory factor analyses, have demonstrated single- [4], two- [5-12], three- [13-16], and four- [17] factor structures. Moreover, recent studies using confirmatory factor analyses have reported three-factor structures. The third factor involved "restlessness" [18], "psychomotor agitation" [19,20], or "negative affectivity" [21-24]. However, most of these factors were highly correlated to anxiety and depression factors. These high correlations suggest that these constructs are essentially identical [18]. Hence, the three-factor models of the HADS may need empirically and theoretically cautious interpretations.

The HADS was originally developed as a tool to be used for a cancer patient sample. In psychiatric research setting several studies reported that depressive symptoms in psychiatric and non-psychiatric samples are of the same quality in terms of the components, and the difference between the two groups is found in terms of illness severity [25]. It remains unclear whether this is true for the HADS. Therefore it is of clinical as well as research importance to confirm if the factor structure of the HADS is the same across psychiatric and non-psychiatric populations.

A third question is the cultural difference of the HADS factor structure. Because most of the past investigations of the HADS factor structure are from the Western countries and it is known that psychological phenomena may vary from one culture to another [26], it is important to examine the HADS factor structure in a non-western culture. To our knowledge, the validity study of the Japanese version of the HADS has yet to be reported.

The main objective of this study is to examine the factor structure of the Japanese version of the HADS in psychiatric outpatient and student populations.

## Methods

### Participants

The data were collected from two groups. The first group consisted of 435 outpatients who attended two psychiatric clinics during a two month period. This group consisted of 157 men, 264 women, and 14 outpatients who did not report their sex. The mean age was 48.0 (SD = 17.0) years. The mean length of treatment was 3.3 (SD = 3.5) years. The median of the length of treatment was 2.0 years. Most of the outpatients (74%) had been attending the clinic for a year or longer, indicating that most outpatients were not in an acute phase of psychiatric illness. Outpatients with dementia, mental retardation, and alcohol or drug abuse were excluded. The second group consisted of 1128 university students of which 431 were men, 696 were women, and one student did not report their sex. The mean age was 20.1 (SD = 3.0) years. A two-way analysis of variance showed that the mean age in the outpatients was significantly higher than the student counterpart (F(1,1544) = 2741.85, *P *< 0.001). However, significant difference between the two sexes, and the sex and group interactions were not found. The sex ratio between the outpatient and student groups did not show differences (chi-squared(1) = 0.12, *P *= 0.732).

Only the participants with complete HADS data were included. Thus, 13 outpatients and 59 students were excluded, but 408 outpatients and 1069 students were analysed.

### Procedure

The existing translation of the HADS Japanese version [27] was used in this study. The questionnaire contained the HADS, items tapping demographic features, and other items that are not reported in this study. The face-sheet provided the aim of this study on an anonymous basis, contact information, as well as the question that encourages a potential respondent to choose either agreement or disagreement to the participation. The questionnaire with an addressed and stamped envelope was distributed in a cross-sectional manner to outpatients as they attended a psychiatric clinic. Each outpatient was asked to complete and return his or her questionnaire by postal mail. The questionnaires were distributed to 1700 outpatients. Of those, 26% were returned. Meanwhile, the questionnaire was distributed to students in psychology classes and returned to the researcher during the class hours. In both settings, the consent was obtained by anonymous submission of the questionnaire marked on the agreement to the participation, and only the data with the consent was included in this study. Thus, each participant's self-determination to participate in the study and the anonymity of response were maintained.

This project was approved by the Ethical Committee of Kumamoto University Graduate School of Medical Sciences, which is equivalent to the Institutional Review Board.

### Statistical analysis

Before beginning a series of factor analyses, we randomly split the sample groups in half (Group 1, n = 739; Group 2, n = 738). The factor analytic procedure allows that the sample in a single study is randomly split in half when the sample size is sufficiently large [28]. An exploratory factor analysis could be performed on one half of the data providing the basis for specifying a confirmatory factor analysis model that can be fit to the other half of the data. Therefore, a plausible model was explored in Group 1 and subsequently cross-validated in Group 2.

To obtain factor solutions in exploratory factor analyses, we used Principal Component Analysis (PCA) as in previous studies. The number of appropriate factors was determined by the eigenvalue above unity [29], the scree test [30], and interpretability of the factors. The substantial threshold of the factor loading in each item was determined as .40 or greater. Confirmatory factor analyses were then performed to identify the optimal model. The maximum likelihood estimation method was adopted to produce standardized parameter estimates. In keeping with common practice, the model fits were evaluated by five indicators: the chi-squared statistic, the Root Mean Squared Error of Approximation (RMSEA) [31], the Comparative Fit Index (CFI) [32], the Tucker-Lewis Index (TLI) [33], and the Akaike Information Criterion (AIC) [34]. The chi-squared statistic is the most common fit test but is almost always statistically significant for models with large samples. A RMSEA of less than .10 indicates an acceptable fit, while less than .05 indicates a good fit. The CFI and TLI values greater than .90 are acceptable fits, while values greater than .95 fit the data well. The TLI is relatively unaffected by sample size. A lower AIC indicates a better fit among a class of competing models. The AIC does not assume a true model, but rather tries to identify the optimal model. Simultaneous multi-group analyses between the outpatients and students and between the two sexes were subsequently conducted to test the factor stability.

We posited that the factor pattern of the HADS was invariant between the outpatient and student groups and between the men and women. This is on the basis of the previous studies reporting the identical components of depressive symptoms in psychiatric and non-psychiatric samples [25]. Therefore, the data was treated as a single dataset, except during subgroup analyses. Statistical analyses were performed using SPSS 10.0 [35] and AMOS version 4.0 [36].

## Results

### Descriptive statistics of the subscales

The mean scores of HADS-A and HADS-D were 7.0 and 6.5, respectively (Table 1). Subgroup analyses indicated that the mean scores of HADS-A and HADS-D in the outpatients were significantly higher than those of the students (HADS-A, t(644) = 7.46; HADS-D, t(610) = 8.87, *P*s < 0.001). Significant main effects of sex, and sex and group interactions were not observed. The cut-off point of the HADS identified possible (8/9) and probable (11/12) cases. As to anxiety, 111 students (10%) and 100 outpatients (25%) were identified as probable cases. As to depression, 77 students (7%) and 96 outpatients (24%) were identified as probable cases. The Cronbach's alpha coefficients were .81 and .76 for HADS-A and HADS-D, respectively. The correlation coefficient between HADS-A and HADS-D was .56 (*P *< 0.001).

**Table 1:** Means and standard deviations of the HADS subscales

	Whole sample	Students	Outpatients
HADS-A			
*Mean*	7.0	6.5	8.3
*SD*	4.0	3.7	4.4
*t (df)*	-	7.46 (644)***	
*Possible cases*	451 (31%)	265 (25%)	186 (46%)
*Probable cases*	211 (14%)	111 (10%)	100 (25%)
HADS-D			
*Mean*	6.5	5.9	8.2
*SD*	4.1	3.7	4.6
*t (df)*	-	8.87 (610) ***	
*Possible cases*	425 (29%)	243 (23%)	182 (45%)
*Probable cases*	173 (12%)	77 (7%)	96 (25%)
Number of samples	1477	1069	408

*** *P *< 0.001. Possible cases were identified by cut-off point = 8/9; Probable cases were identified by cut-off point = 11/12.

### Factor structure

Principal component analysis with a Promax rotation extracted two factors with a moderate correlation in the people in Group 1. The first five eigenvalues were 4.85, 1.43, .98, .97, and .82. A scree test supported the two-factor solution. These factors represented anxiety and depression (Table 2). All items, except for items 6, 7, and 10, constituted the appropriate factors. Items 6 and 7 loaded on neither factor and showed certain degree of dual loadings, but item 10 indicated only a low contribution to the depression factor.

**Table 2:** Factor loadings of the HADS items in Group 1

HADS item	F1	F2
HADS-A		
Item 1: feeling of tension	**0.73**	-0.11
Item 3: frightened feeling	**0.72**	0.03
Item 5: worrying thoughts	**0.69**	0.13
Item 7: relaxed feeling	0.26	0.39
Item 9: butterflies in stomach	**0.68**	0.00
Item 11: restless feeling	**0.55**	-0.07
Item 13: feeling of panic	**0.79**	0.02
HADS-D		
Item 2: enjoyment	-0.19	**0.86**
Item 4: laughter	-0.04	**0.81**
Item 6: cheerful feeling	0.35	0.23
Item 8: feeling slowed down	0.19	**0.42**
Item 10: lost interest in appearance	0.09	0.30
Item 12: look forward to things	-0.01	**0.76**
Item 14: enjoyment of book/radio/TV	0.05	**0.70**
Eigenvalue	4.85	1.43
Subscale correlation	.51	

Bold face indicates loadings with absolute values of 0.40 or more.

**Figure 1:**
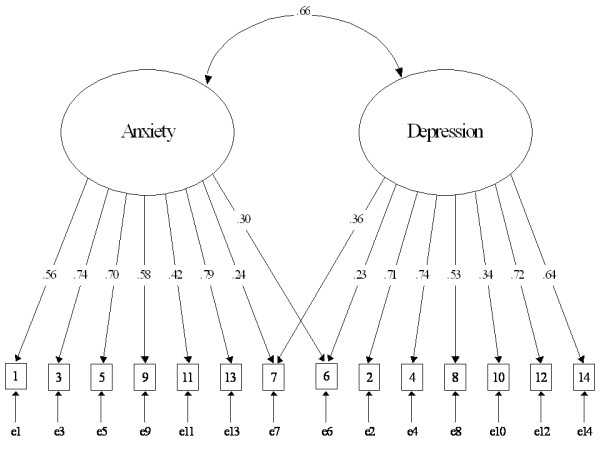
**Factor structure of the HADS**. Boxes represent observed variables; Ellipses represent latent variables; Single-headed arrows represent regression weights; Double-headed arrow represents correlation.

Using the data of Group 2, a confirmatory factor analysis examined the models refined in this study as well as in the previous studies. The current model defined in this study is derived from the results of the exploratory factor analyses. This model consists of the correlated anxiety and depression factors, and allows items 6 and 7 to each load on both the anxiety and depression factors. Item 10 only loads on the depression factor due to the low contribution to the anxiety factor described above. Thus, in the current model the anxiety factor consists of all the original anxiety items and item 7, but the depression factor consists of all the original depression items and item 6. Table 3 shows the model fit indexes among the competing models in Group 2. Of these models, the current model indicated the best fit to the present data. The chi-squared statistic was 187.45 (d.f. = 74, *P *< 0.001). The RMSEA, CFI, and TLI were .046, .963, and .955, respectively. The AIC was 249.445, which was lowest among the models. Figure 1 shows the factor loadings of the current model. Although items 6, 7, and 10 indicated low contributions, all factor loadings were significant (Ps < 0.001). Upon assuming the third factor consisting of the items 6, 7, and 10, the model showed poorer fits (AIC = 365.723). Upon deleting either items 6, 7, or 10, the models once again showed poorer fits (AIC = 419.287, 473.140, and 324.343, for the items 6, 7, and 10, respectively). In order to confirm the robustness of the results, we reversed the order of the analyses. Thus, we performed an exploratory factor analysis using the Group 2 data and then used the Group 1 data for a confirmatory factor analysis (Table not shown). The results obtained were virtually the same. A simultaneous confirmatory factor analysis between the outpatients and student groups was conducted. Table 4 shows the absolute indexes of the goodness-of-fit in the modified oblique models, Models A, B, and C. Model A was the baseline model used to test the common factor pattern, while the magnitude of the factor loadings was allowed to vary. This model provided an equally good fit for the data across the two groups with .938, .930, and .038 for CFI, TLI, and RMSEA, respectively. Model B assumed that the corresponding factor loadings between the two groups were equal. When all factor loadings except for the factor covariance was constrained, the model fitness of Model B was significantly poorer than Model A. Therefore, we released the factor loadings constraints using the modification indices until the best-fit model was determined. Although half of the factor loadings in the anxiety items were imposed constraints, only two factor loadings in the depression items could be constrained. The items tapping anhedonics (items 2, 4, 12, and 14) in the outpatients showed higher factor loadings than those in the students. Model C was the same as Model B except that the respective common factor variance for the two groups was assumed to be equal. When the factor covariance was constrained, the model fit slightly decreased (AIC = 601.269), but remained acceptable. All the chi-squared statistics did not indicate significant increments between Model A and B (chi-squared(6) = 7.55, *P *= 0.273), and between A and C (chi-squared(7) = 10.82, *P *= 0.146). The subgroup analysis between men and women showed complete invariance; the factor pattern, factor loadings, and common factor variance were constrained, providing acceptable to excellent fits. All the chi-squared statistics did not indicate significant increments between Model A and B (chi-squared(16) = 13.19, *P *= 0.659), and between A and C (chi-squared(17) = 13.21, *P *= 0.722).

**Table 3:** Fit indexes of the current and proposed models in Group 2

Model	N. of factor	Chi-squared (d.f.)	RMSEA	CFI	TLI	AIC
Razavi et al. (1990)	1	241.832 (77)	.108	.864	.839	297.832
Zigmond et al. (1983)	2^a^	261.998 (77)	.115	.847	.819	317.998
Moorey et al. (1991)	2^b^	231.387 (76)	.053	.949	.939	289.387
Current study	2	**187.445 (74)**	**.046**	**.963**	**.955**	**249.445**
Dunbar et al. (2000)	3	211.682 (72)	.051	.955	.943	277.682
Caci et al. (2003)	3^c^	578.375 (74)	.096	.837	.799	640.375
Leung et al. (1993)	3	218.079 (74)	.051	.953	.943	280.079
Friedman et al. (2001)	3^d^	240.598 (74)	.055	.946	.934	302.598

All chi-squared statistics were significant at *P *< 0.001. ^a^Original two factors. ^b^Two factors were correlated. ^c^Three factors consisting of all 14 items. ^d^Three factors were correlated.

**Table 4:** Fit indexes of the invariance of the HADS across the subgroups

	Chi-squared(df)	RMSEA	CFI	TLI	AIC
Outpatients vs. students					
Model A	508.444 (162)	.038	.938	.930	604.444
Model B	515.996 (168)	.037	.938	.932	599.996
Model C	519.269 (169)	.037	.937	.932	601.269
Men vs. women					
Model A	498.638 (162)	.038	.943	.936	594.638
Model B	511.829 (178)	.036	.943	.942	575.829
Model C	511.852 (179)	.036	.944	.943	573.852

Model A is factor pattern invariance; Model B is factor loading invariance; Model C is strong factorial invariance. All chi-squared statistics were significant at *P *< 0.001.

## Discussion

The aim of the present study is to examine the factor structure of the HADS using Japanese psychiatric outpatient and student populations. We demonstrated that the HADS consists of two factors, which represent anxiety and depression with moderate correlations. The factor structure refined by exploratory factor analysis includes the error variance due to measurement error and a random component in the measured phenomenon. In contrast, confirmatory factor analysis allows the error variables independent from the observed variables. Thus, the factor structure examined by the confirmatory factor analysis stringently excludes the influence of error variance. When both methodologies support a two-factor structure, the model shown in the exploratory factor analysis provides a stronger validity than the result from the confirmatory factor analysis because the two-factor structure is thoroughly robust despite the errors. Thus, the result in this study is consistent with earlier exploratory studies [5-12].

The two-factor structure in this study is empirically derived. The anxiety and depressive symptoms observed in psychiatric evaluation entail both state and trait aspects. The trait aspects are partly composed of negative affective personality. For example, anxiety, depression, and neuroticism are partly explained by a common genetic factor [37,38]. These reports appear to explain the facts that the two distinct symptoms are frequently comorbid. Neuroticism accounts for the comorbidity between anxiety and depressive disorders [39]. This type of personality, especially negative affective temperament, can be considered either as a personality trait or as a trait aspect of anxiety and/or depressive symptoms [40]. The tripartite model [41] assumes that the negative affectivity shared by anxiety and depression involves a trait-like construct, including neuroticism. This is theoretically sophisticated. However, when empirical data show high correlations between negative affectivity and anxiety or depression, the constructs of negative affectivity should be reduced to anxiety or depressive symptoms. Barbee [42] noted that symptom-based diagnoses are the best alternative when the aetiology of anxiety and depressive disorders is not substantially determined. Thus, the HADS tapping anxiety and depression symptoms are reasonable in terms of factor structure.

The model in this study is consistent for all the subgroups. As expected, the factor pattern of the HADS in this study is same across the outpatient and student groups. The major difference between the two groups is the severity of anxiety and depression. In addition, this model completely coincides between men and women. Several differences between the outpatient and student samples were observed in the factor loadings. In this study, half the factor loadings of the anxiety items could be constrained, suggesting that a certain part of psychic anxiety is invariant across the outpatient and student samples. One possible explanation is that the HADS excludes somatic symptoms. General Anxiety Disorder often accompanies anxiety or panic attacks presented as dyspnea, tachysystole, and sweating [43]. These somatic symptoms of anxiety may be a clear difference between the outpatient and student samples. The other possibility is that most outpatients in this study are in the chronic phase and their anxiety symptoms had been vastly improved through long-term treatment. Although the mean scores of HADS-A were significantly higher in the outpatients, the factor loadings of mild anxiety may be more similar to those of the students.

In contrast, few factor loadings of the depression items could be constrained. The difference between the outpatient and student groups is particularly obvious in the items that are assumed to reflect anhedonics. This result suggests that the effect of the depression construct on each item is different between the two groups. One plausible explanation is that the HADS-D focuses on anhedonic symptoms. Anhedonics are the core symptoms of Major Depressive Disorder [44]. The difference in factor loadings of the depression items may partly depend on the severity of depression. Thus, the HADS-D may be more reliable in a psychiatric sample compared to a non-psychiatric sample.

This study was conducted on the outpatient and student samples. It remains possible that different structures exist for different target populations. Factor analytic studies frequently reported that the constructs can vary in different subgroups of the sample [45-47]. When people with physical illness were included in our sample, the construct may vary. For instance, people with cancer mostly suffer pain, fatigue, and insomnia [48,49]. Previous studies indicated that cancer-related pain was linked to anxiety relative to depression [50-52], and that cancer-related fatigue/insomnia deteriorated depression [53]. The influence of such physical symptoms on the factor structure of the HADS has not been substantially identified. Further investigation is required.

Several items need to be carefully examined. In our two-factor model, items 6 and 7 each indicated dual loadings for anxiety and depression factors. Among previous studies, which have reported two-factor solutions, item 7 ("I can sit at ease and feel relaxed") have shown high factor loadings for either the anxiety [1] or depression factor [8]. This discrepancy may stem from the ambiguous wording. Item 7 simultaneously refers to psychomotor agitation ("cannot sit at ease") and inner tension or anhedonia ("cannot feel relaxed"), which may cause the dual loading in this study. To clarify the target construct, this double-barrel question should be divided into two sentences in future revisions [54]. Item 6 also indicates dual loading. This finding may be specific to the Japanese population. Previous studies have consistently reported that item 6 constitutes a depression factor with moderate loading [1,8,13,18,22]. Although the language equivalence of the Japanese version of HADS is well established [27], the response bias changes the basic nature of the depression item to an anxiety item. The item 6 ("I feel cheerful") when translated into Japanese connotes the shift of the mood from its cheerful comfortable state. It may suggest, to some extent, irritability and feeling upset in addition to despondency. This may cause a response option with negative expression. Further studies on the response bias of the Japanese version of the HADS are needed.

In addition, item 10 needs to be more closely examined in order to determine the consistency with the other depression items. Item 10 in this study had low contributions in both the exploratory and confirmatory factor analyses. This is congruent with the previous studies [18]. The item asking personal appearance may be influenced by a construct other than depression, such as interpersonal attraction and/or social desirability. Thus, further investigation is necessary to identify the confounding factors of item 10.

Despite these minor shortages, the scoring system of the HADS should adhere to the original instructions by Zigmond and Snaith [1]; the HADS-A and HADS-D subscales should each be comprised of the original seven items. The confirmatory factor analyses in this study suggest that all items show a substantial contribution to the fitness of the current model. Although the item 6 showed higher loadings on the anxiety factor and the item 7 indicated higher loading on the depression factor, these inappropriate loadings appear to be stemmed partly from the wording issues previously mentioned. The revision of the HADS should be started from such language issues in advance of the rescoring. In the original scoring system, however, the two of the depression items (item 6 and 10) may undermine a precise evaluation of depressive level as suggested by the low contributions to the depression factor. Indeed the Cronbach's alpha coefficient of the HADS-D was lower than that of the HADS-A in this study. Therefore, it should be noted that the validity and reliability of the HADS-D subscale is inferior to the HADS-A subscale in the current Japanese version of the HADS.

This study has some limitations. First, our sample does not include people with bodily diseases. The HADS was originally developed to detect anxiety and depression in a hospital setting [1]. The influence of somatic symptoms on the factor structure of the HADS is still unclear. Further research that compares different types of medically ill patients should determine the usability of the HADS. Second, the low response rate in the outpatient group may involve a response bias for the questionnaire. Non-respondents may partly include outpatients in an acute phase of psychiatric illness, while most of the respondents were in a chronic phase. Thus, the findings in this study should be confined to relatively improved symptoms of anxiety and depression in the outpatients. Third, this study collected cross-sectional HADS data. Thus, the factor stability over time remains unclear. Previous studies have reported that early onset of anxiety disorders is linked to subsequent depression [55,56]. These changes in the symptoms during a clinical course may influence factorial validity. A longitudinal research study would allow the temporal stability of the HADS to be examined. Finally, the construct overlap between the HADS and the other assessment instruments was not examined. The HADS emphasizes psychic symptoms of autonomic anxiety and anhedonic depression, while other scales (e.g., Beck Depression Inventory [57] and State-Trait Anxiety Inventory [58]) tap broader components such as helplessness and somatic symptoms of anxiety and depression. The convergent validity of the HADS should be confirmed in relation to the other anxiety and depression scales. Joint factor analysis may provide evidence of item overlap in broader constructs of anxiety and depression across instruments.

## Conclusion

Our results empirically support the correlated two-factor structure of the HADS in Japanese outpatient and student populations. The HADS is a factorially valid and reliable instrument with a robust structure in terms of psychiatric as well as medical settings.

## Competing interests

The authors declare that they have no competing interests.

## Authors' contributions

TM and TK planned the study. HK and RK collected data from student populations. HM and KO collected data from a clinical population. HI gave advices and comments from a clinical perspective. TM wrote the manuscript.
